# Evidence for Variation in the Genetic Basis of Sex Determination in Brook Stickleback (*Culaea inconstans*)

**DOI:** 10.1002/ece3.70955

**Published:** 2025-02-12

**Authors:** Grace C. Pigott, Massa Abo Akel, Malcolm G. Q. Rogers, Marin E. Flanagan, Erica G. Marlette, Matthew J. Treaster, Shannon K. Fox, Shaugnessy R. McCann, Catherine L. Peichel, Michael A. White, Daniel L. Jeffries, Jonathan A. Mee

**Affiliations:** ^1^ Department of Biology Mount Royal University Calgary Alberta Canada; ^2^ Department of Genetics University of Georgia Athens Georgia USA; ^3^ Division of Evolutionary Ecology, Institute of Ecology and Evolution University of Bern Bern Switzerland

## Abstract

The genetic basis of sex determination is typically conserved within species if not within broader lineages. For example, within the stickleback family (Gasterosteidae), *AmhY* has been identified as a master sex‐determination (MSD) gene in multiple species across two genera. By contrast, the existence of within‐species variability in the genetic basis of sex determination is not frequently observed but provides an opportunity to understand the evolution and turnover of sex determination systems. In this study, we investigated the consistency with which *AmhY* is involved in sex determination across 610 individuals from five brook stickleback ( *Culaea inconstans* ) populations. We designed a PCR‐restriction enzyme assay to identify the presence of *AmhY* in each individual and recorded sexual morphology in each individual in the field at the time of capture. We found that the genetic sex (presence/absence of *AmhY*) did not match the field‐determined phenotypic sex in up to 44% of individuals within a population. This variation in the genetic basis of sex determination in brook stickleback suggests that the mechanism of sex determination in this species is likely more complex than thought when *AmhY* was first implicated and may still be evolving. Such within‐species variation provides an opportunity to further investigate how and why transitions in sex‐determination mechanisms occur.

## Graphical abstract

The genetic basis of sex determination is typically conserved. In brook stickleback, we found that the genetic sex did not match the phenotypic sex in up to 44% of individuals per population. Within‐species variation in genetic sex determination provides an opportunity to investigate how and why transitions in sex‐determination mechanisms occur.

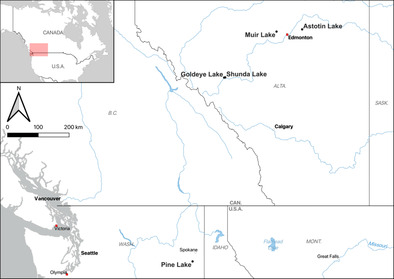

## 1 Introduction

Sex determination mechanisms are diverse and complex across the Tree of Life, and no less so among vertebrates. Genetic sex determination, where an individual's genotype determines its sex, is common among vertebrates, although the mechanisms and genes underlying genetic sex determination are, in turn, also diverse and complex (Tree of Sex Consortium 2014). One mechanism of genetic sex determination involves a master sex‐determination (MSD) gene, which initiates the differentiation between females and males during development. MSD genes have been identified in both male heterogametic (XY) and female heterogametic (ZW) systems (Volff et al. 2007; Yoshimoto et al. 2008; Smith et al. 2009; Eggers and Sinclair 2012; Chen et al. 2014). Some MSD systems are highly conserved, with the same gene underlying sex determination in every species in a lineage (Foster and Graves 1994). An example of a conserved MSD gene is the Y‐specific *Sry* gene that is homologous in the male heterogametic system across almost all mammals (Foster and Graves 1994; Hughes, Lagunas‐Robles, and Campbell 2024). It is also common for several species or lineages to share the same MSD gene via convergent evolution (Bull 1983; Charlesworth 1996; Ming, Bendahmane, and Renner 2011). For example, *Dmrt1* (or a paralog) has been recruited independently to act as the primary sex‐determination gene in the African clawed frog ( *Xenopus laevis* ; Yoshimoto et al. 2008), chicken ( *Gallus gallus* ; Smith et al. 2009), medaka ricefish ( *Oryzias latipes* ) (Nanda et al. 2002), and the smooth tongue sole ( *Cynoglossus semilaevis* ) (Chen et al. 2014). In other lineages, MSD genes have been recruited independently in closely related species. For example, lineages with independent recruitment of MSD genes in closely related species include fish in the genus *Oryzias* (Myosho et al. 2012; Nagahama et al. 2002; Nanda et al. 2002) and fish in the stickleback family (Teleostei: Gasterosteidae; Jeffries, Mee, and Peichel 2022).

Sticklebacks are an interesting model system for studying the evolution of sex determination due to their sex chromosome diversity (Ross et al. 2009; Dixon, Kitano, and Kirkpatrick 2019; Natri, Merilä, and Shikano 2019; Sardell et al. 2021; Yi et al. 2024). Among sticklebacks, the anti‐Muellerian hormone gene, *Amh*, has been recruited at least two times independently as the sex‐determination gene (Jeffries, Mee, and Peichel 2022). One of these systems arose approximately 22 million years ago in the ancestor of the genus *Gasterosteus*, in which *Amh* (ancestrally on Chr08) was duplicated to chromosome 19, which then became an XY sex chromosome pair. In all three species of the genus, the duplicate copy of *Amh* (*AmhY*) is found on the Y chromosome, but not the X chromosome (Peichel et al. 2020; Sardell et al. 2021; Dagilis et al. 2022). The second independently evolved *Amh* system in stickleback was recently described in brook stickleback ( *Culaea inconstans* ). Jeffries, Mee, and Peichel (2022) found evidence that the ancestral chromosome 8 copy of *Amh* (henceforth referred to as *Amh08*) has again duplicated, this time to chromosome 20 (referred to as *AmhY*), and this duplicate may now function as the sex‐determination gene in this species. Importantly, *Amh08* and *AmhY* differ by only four single nucleotide polymorphisms (SNPs), suggesting a relatively recent origin of *AmhY* in brook stickleback independent of the *Gasterosteus AmhY* origin (Jeffries, Mee, and Peichel 2022). This hypothesis is also supported by the absence of extensive differentiation between the brook stickleback X and Y chromosomes (chromosome 20), which is a hallmark of young sex chromosomes (Charlesworth, Charlesworth, and Marais 2005; Furman et al. 2020).

The study by Jeffries, Mee, and Peichel (2022) was based on a whole‐genome sequence analysis of 84 individuals from Shunda Lake in Alberta, Canada, and an F_1_ Lab cross between a female from Fox Holes Lake in Northwest Territories, Canada, and a male from Pine Lake in Alberta, Canada, using the *P. pungitius* reference genome. In the study by Jeffries, Mee, and Peichel (2022), all males carried *AmhY*, but nine of the samples identified as female in the field were also found to be carrying *AmhY* (Jeffries, Mee, and Peichel 2022). The mismatch between field‐identified and genetic sex in these samples was thought to be due to error in the field, such as an incorrect inference that an individual was female based on the absence of male nuptial coloration without any observation of female characteristics (such as mature ova). These mismatched samples were removed from subsequent analyses in the study by Jeffries, Mee, and Peichel (2022). It is possible, however, that these individuals were truly female and that *AmhY* is not fully penetrant in its function as a sex determination gene.

The initial aim of this study was to develop a genetic assay for sex identification in brook stickleback using sequence information from the *AmhY* locus. We designed a PCR and restriction enzyme assay to evaluate the presence‐absence of *AmhY* among individuals with reliably identified sex (e.g., with records of observations at the time of capture of eggs or clear male nuptial coloration). We initially discovered a high frequency of mismatches between the presence‐absence of *AmhY* and reliably identified phenotypic sex. Hence, our research goal shifted to describing and potentially explaining the variation in the genetic basis of sex determination in brook stickleback. We therefore assayed the presence‐absence of *AmhY* in 610 individuals from four brook stickleback populations in Alberta, Canada, and one population in Washington, USA. Our results suggest the existence of within‐species variability in the genetic basis of sex determination, which is not frequently observed. Thus, the results of this study are of great value to understanding not only the brook stickleback sex‐determination system but, more broadly, the evolution and turnover of sex determination systems across the Tree of Life.

## 2 Methods

### 2.1 Sample Collection

**FIGURE 1:**
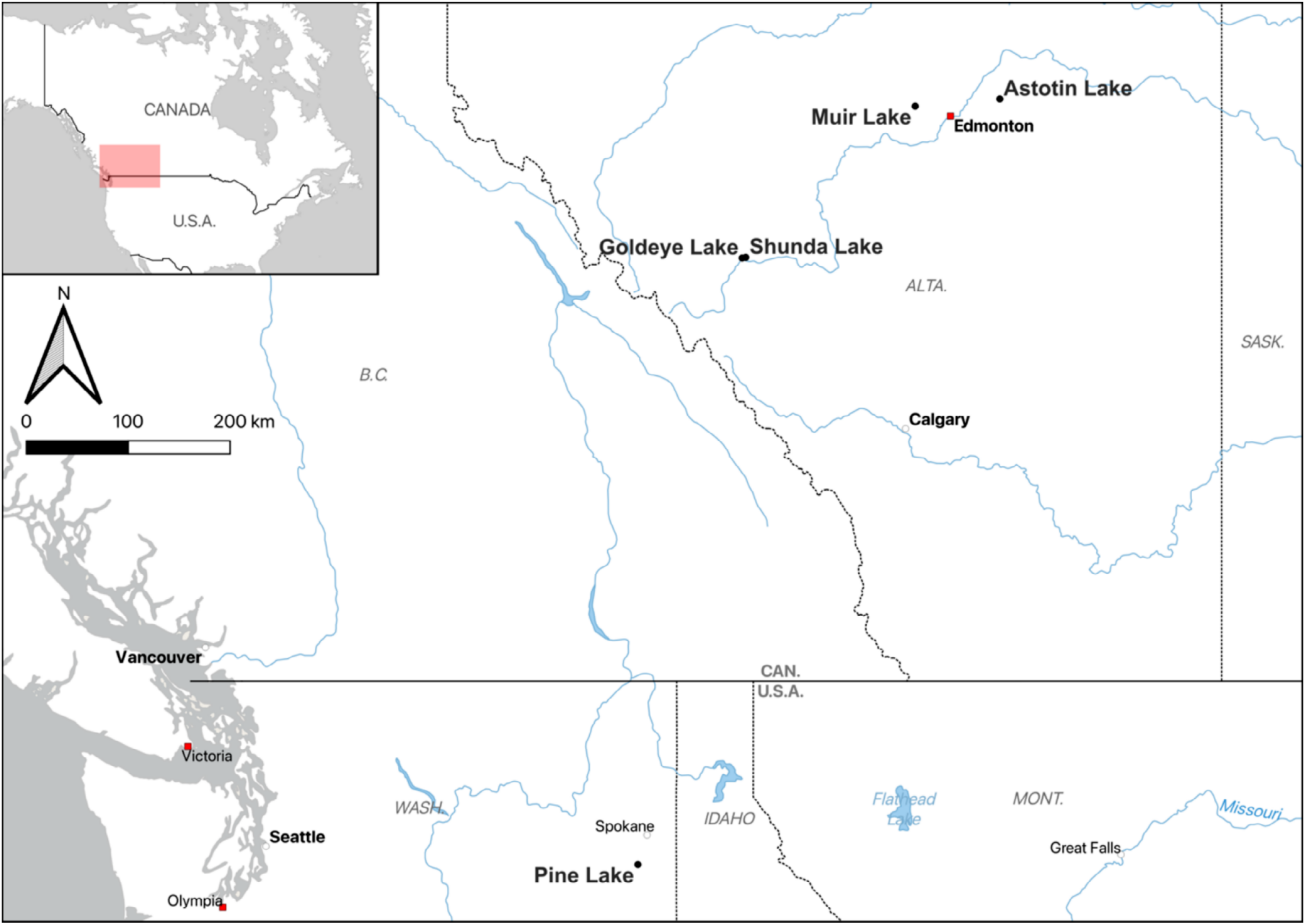
Map showing the locations of brook stickleback populations in Alberta, Canada, and Washington, USA, sampled for this study. The pink shading in the inset map shows the area displayed in the larger‐scale map. The map was made with QGIS using Natural Earth Data.

Brook stickleback were collected from four populations in Alberta, Canada (Figure 1): Astotin Lake (UTF‐8 encoded WGS84 coordinates: 53.679907 latitude, −112.861954 longitude), Goldeye Lake (52.447012, −116.191621), Muir Lake (49.816676, −97.220355), and Shunda Lake (52.453899, −116.146192). Brook stickleback were also collected from one recently introduced invasive population (Scholz et al. 2003; Gunselman 2017) in upper and lower Pine Lake within the Turnbull National Wildlife Refuge (Spokane County, Washington, USA; lower site: 47.409078, −117.538096; upper site: 47.412611, −117.538679; Figure 1). Note that this Pine Lake population is different than the Alberta population used for genetic mapping in Jeffries, Mee, and Peichel (2022). Unbaited minnow traps (5 mm mesh) were placed adjacent to submerged aquatic vegetation or under overhanging vegetation at a depth of 0.5‐2 m along the shorelines of the lakes for 1–12 h. Each brook stickleback used for this study was euthanized using 0.5 mL of clove oil per 2000 mL of lake water for the Alberta populations (Javahery, Nekoubin, and Moradlu 2012) or 0.05% MS‐222, buffered to neutral for the Pine Lake population. Caudal fin clips were preserved in 95% ethanol, and the bodies were preserved in 70% ethanol. For the Pine Lake population, whole fish were preserved in 95% ethanol for shipping and then transferred to 75% ethanol. Samples were collected during the breeding season (June and early July) when sex can be recorded in the field. Females were identified by the presence of eggs (e.g., released with gentle pressure to the abdomen during handling), the presence of a distended abdomen (without an abdominal cestode parasite, verified via dissection), and/or the presence of an egg mass or ovaries (visible via ventral dissection). Males were identified by presence of dark nuptial coloration (which is substantially different from female nuptial coloration; McLennan 1995) and/or the presence of testes (visible via ventral dissection). We confidently identified sex in a total of 546 individuals from Astotin Lake, Goldeye Lake, Muir Lake, and Shunda Lake among samples collected in 2017, 2019, 2020, 2022, 2023, and 2024 as well as 64 individuals from Pine Lake collected in 2023 (Table 1). Samples were collected from Alberta under fisheries research licenses issued by the Government of Alberta and, in the case of Astotin Lake, by Parks Canada. The Mount Royal University Animal Care Committee approved collection methods and the use of animals in research (Animal Care Protocol ID 101029 and 101795). The Pine Lake samples were collected under a research and monitoring special use permit issued by the Turnbull National Wildlife Refuge (permit number 13560‐23‐010). Collection of Pine Lake individuals was approved by the University of Georgia Animal Care and Use Committee (protocol A2021 07‐031‐A11). For the samples from Alberta, DNA was extracted from fin clips using a Qiagen DNEasy Blood and Tissue kit (Cat. No. 69506). For the Pine Lake samples, DNA was extracted from fin clips using a HotSHOT DNA extraction protocol (Archambeault et al. 2020) adapted from Meeker et al. (2007).

**TABLE 1:** Summary of the field‐determined sex of all 610 samples from Astotin Lake, Goldeye Lake, Muir Lake, Shunda Lake, and Pine Lake that were collected during 2017, 2019, 2020, 2022, 2023, and 2024. Individuals with unknown phenotypic sex were not analyzed in this study.

Lake	Year	Male	Female
Astotin	2022	27	76
Goldeye	2020	4	16
	2023	8	11
	2024	13	19
Muir	2017	46	49
	2019	49	11
Shunda	2017	39	56
	2019	29	30
	2022	25	25
	2023	7	6
Pine	2023	33	31

### 2.2 Genotyping Assay Design

Of the four SNP differences between the ancestral and derived *Amh* genes on chromosomes 8 and 20, respectively (Jeffries, Mee, and Peichel 2022), one SNP lies within a BsaAl restriction enzyme cut site such that *Amh08* has the restriction site and *AmhY* does not (see supporting information for fasta formatted *Amh* sequence information from Jeffries, Mee, and Peichel 2022). For the Albertan samples, we used the Integrated DNA Technologies PrimerQuest tool to design PCR primers that flanked the BsaAl restriction enzyme cut site, with a fragment of length of 230 bp. The primer sequences were: 5′‐GTGGTCAATCACCTCCACTATC‐3′ and 5′‐ACAAATGCGGGCTGAAGA‐3′. After digestion of the *Amh08* gene with BsaA1, we expected two fragments of 143 and 87 bp. As the restriction enzyme cut site is disrupted by a SNP in *AmhY*, amplicons from this gene will not be digested. Individuals that do not carry *AmhY* will thus produce only two fragments at 143 and 87 bp, while individuals that carry *AmhY* and *Amh08* will show three fragments of 230 bp (undigested *AmhY* amplicons), 143, and 87 bp (digested *Amh08* amplicons). For Pine Lake samples, PCR primers of this region were designed in Geneious using a modified version of Primer3. The primer sequences were 5′‐CTTCCTCCTGCTGAAGGCC‐3′ and 5′‐CACCCGCACTCTTTGGCC‐3′ which produced a 381 bp amplicon. After digestion with BsaAI, the amplicon from *Amh08* would produce two fragments at 244 and 137 bp.

### 2.3 Genotyping Assay Conditions

For the Albertan samples, the PCR reaction mixture was designed to have a final volume of 20 μL. The reaction mixture contained 1 μL of genomic DNA with concentrations ranging from 1.17–372 ng/μL, primers at a final concentration of 0.5 μM each, dNTPs at a concentration of 0.2 mM each, two units of Platinum Taq DNA Polymerase (Invitrogen Cat. No. 10966018), its associated 10× PCR buffer (without magnesium), and MgCl_2_ at a final concentration of 1.5 mM. Using an Eppendorf Vapoprotect Mastecycler Pro, the initial pre‐heating and denaturing step was set at 95°C for 2 min, which was followed by 35 cycles of denaturing (95°C for 30 s), primer annealing (52°C for 30 s), and elongation (72°C for 30 s). The final elongation step was set at 72°C for 5 min. Post elongation, samples were stored at 4°C until the restriction enzyme digest was performed.

PCR reactions for the Pine Lake samples were performed in a total volume of 20 μl using 0.8 μL of genomic DNA, primers at a final concentration of 0.2 μM each, dNTPs at a concentration of 0.2 mM each, and 0.5 units of DreamTaq DNA polymerase (Thermo Scientific Cat. No. EP0705), and the associated 10× DreamTaq Green Buffer with 20 mM MgCl_2_. Using an Analytik Jena Biometra TOne, the initial pre‐heating and denaturing step was set at 95°C for 2 min, which was followed by 35 cycles of denaturing (95°C for 30 s), primer annealing (60°C for 30 s), and elongation (72°C for 30 s). The final elongation step was set at 72°C for 5 min. Post elongation, samples were stored at 4°C until the restriction enzyme digest was performed.

**FIGURE 2:**
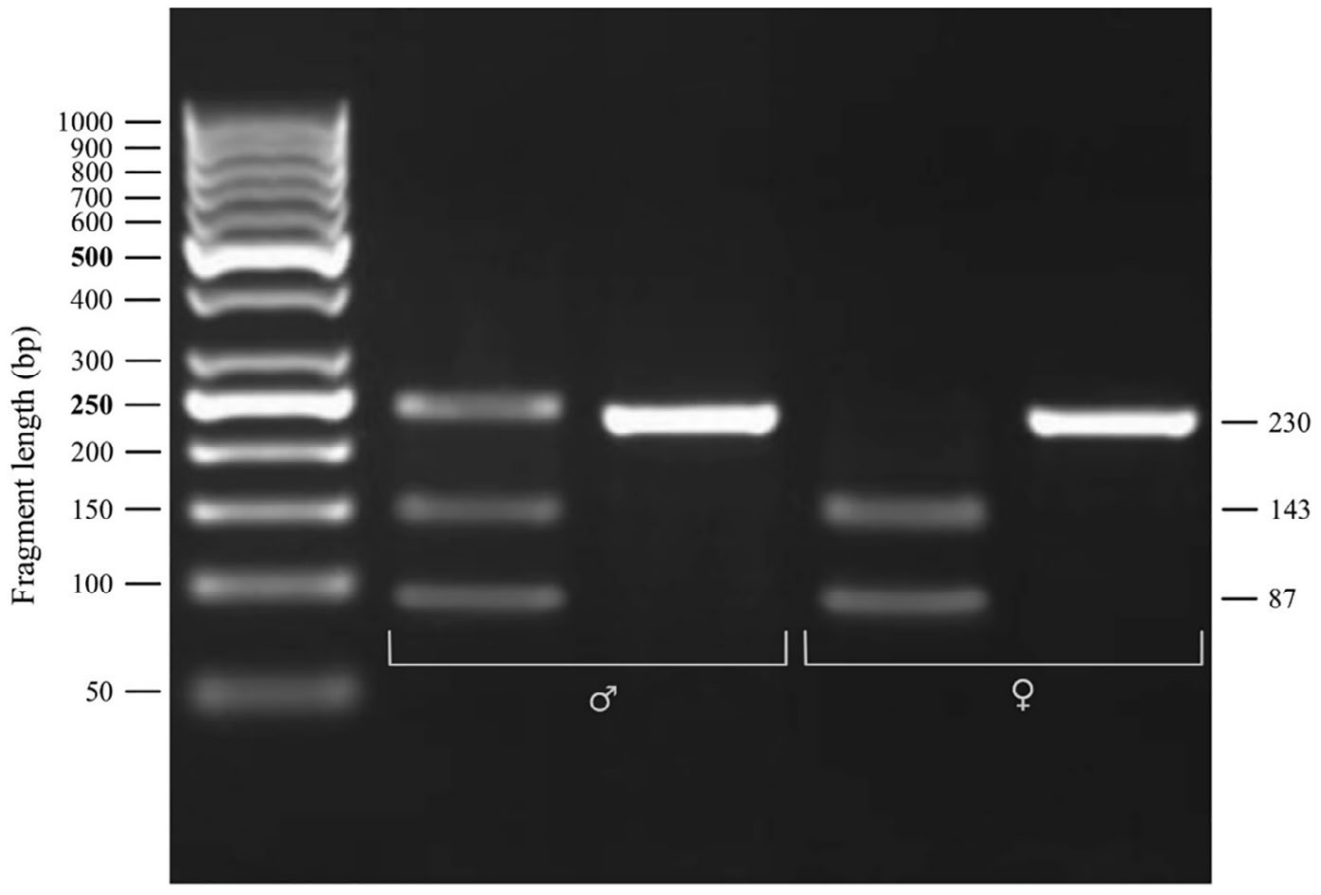
Image of the PCR‐restriction enzyme *AmhY* assay results for two samples from Goldeye Lake collected in 2024. The sample on the left is a male (♂), visualizing its digested (left) and undigested (right) PCR fragments. The sample on the right is a female (♀), visualizing its digested (left) and undigested (right) PCR fragments. The *AmhY* gene copy does not have a BsaA1 restriction site and shows up as a 230 bp fragment in the digested PCR product (e.g., in the male depicted here).

The contents of the BsaA1 digestion reaction mixture (30 μL final volume) followed the manufacturer's specifications (New England Biolabs, Cat. No. R0531S). The PCR products were incubated in a water bath at 30°C for 90 min with the BsaAl restriction enzyme to ensure the restriction reaction had been completed. The digested fragments were visualized using a 1.5% agarose gel with ethidium bromide staining (Figure 2). To determine the lengths of the fragments, a 50 bp ladder (Thermo Scientific) was loaded along with 5 μL of undigested PCR product and 15 μL of the corresponding digest product. Gel electrophoresis was performed using Bio‐Rad PowerPac Basic at 100 V for 40 min. For the Pine Lake samples, 5 μL of PCR product was digested with BsaAI in a 20 μL reaction following the manufacturer's specifications (New England Biolabs Cat. No. R0531S). The restriction digests were incubated at 37°C for 30 min and were analyzed on a 2% agarose gel with SYBR Safe DNA Gel Stain (Invitrogen Cat. No. S33102). 2 μL of GeneRuler 1 kb Plus DNA Ladder (Thermo Scientific Cat. No. SM1331) was loaded along with 20 μL of each digest product. Gel electrophoresis was performed using a VWR Electrophoresis Power Supply at 140 V for 30 min.

## 3 Results

We used the PCR‐restriction enzyme assay to genotype the *Amh* locus in 610 individuals (Table 1). In all five lakes, some individuals produced three bands, and some individuals produced two bands. Hence, it appears that *AmhY* is present in all five lakes and that it is the same duplication on chromosome 20 as described in Jeffries, Mee, and Peichel (2022). But, as described above, the assay‐determined sex and the field‐determined sex were mismatched for several individuals (Table 2). Astotin Lake, Goldeye Lake, Muir Lake, Shunda Lake, and Pine Lake had total mismatch proportions of 44%, 11%, 37%, 16%, and 11%, respectively. Males collected from Shunda Lake had 99% alignment (only one mismatch) between field‐determined and assay‐determined sex. A similar result was found in Goldeye Lake, which is approximately 3 km west of Shunda Lake in the same watershed, where all but one field‐determined male was found to carry *AmhY*. However, in Shunda Lake and Goldeye Lake, 28% and 15% of the field‐determined females, respectively, were found to carry *AmhY*. In Astotin Lake, which had the highest rate of mismatch, 30% of field‐determined males lacked *AmhY* and 49% of field‐determined females carried *AmhY*. In Muir Lake, 35% of field‐determined males lacked *AmhY* and 42% of field‐determined females carried *AmhY*. In the invasive Pine Lake population, 21% of males lacked *AmhY* and 0% of females carried *AmhY*.

**TABLE 2:** Summary of the proportion of mismatch between field‐determined phenotypic sex and assay‐determined genotypic sex for males and females in each population and each year sampled for this study.

Lake	Year	Male mismatch	Female mismatch
Astotin	2022	29.63% (*n* = 8/27)	48.68% (*n* = 37/76)
Goldeye	2020	25.00% (*n* = 1/4)	25.00% (*n* = 4/16)
	2023	0.00% (*n* = 0/8)	18.18% (*n* = 2/11)
	2024	0.00% (*n* = 0/13)	5.26% (*n* = 1/19)
Muir	2017	39.13% (*n* = 18/46)	36.73% (*n* = 18/49)
	2019	30.61% (*n* = 15/49)	63.64% (*n* = 7/11)
Shunda	2017	2.56% (*n* = 1/39)	25.00% (*n* = 14/56)
	2019	0.00% (*n* = 0/29)	23.33% (*n* = 7/30)
	2022	0.00% (*n* = 0/25)	44.00% (*n* = 11/25)
	2023	0.00% (*n* = 0/7)	16.67% (*n* = 1/6)
Pine	2023	21.21% (*n* = 7/33)	0.00% (*n* = 0/31)

To explore whether our assay results are reflective of a true biological pattern, as opposed to experimental error (e.g., a faulty assay), we compared our results directly to those in the Jeffries, Mee, and Peichel (2022) study, focusing on the 84 samples from Shunda Lake that were analyzed in that study. All the males (*n* = 46) from the Jeffries, Mee, and Peichel (2022) study were positive for *AmhY* in our assay. In contrast, 20% (8) of the females (*n* = 38) in the Jeffries, Mee, and Peichel (2022) study were also positive for *AmhY* in our assay. The female samples with mismatched sex in our assay were the same samples excluded from the analysis in Jeffries, Mee, and Peichel (2022), due to the assumption of erroneous sex identification. We subsequently confirmed the field‐based sex identification of these eight individuals as females using our field notes and revisiting the preserved samples. The eight females with *AmhY* according to our assay also had an excess of putatively male‐specific k‐mers in a preliminary analysis of the whole‐genome sequence data (unpublished results), suggesting that they indeed carry the entire duplicated region.

Although we do not have sequencing data for the four remaining populations, it is unlikely that mismatches between field‐determined sex and assay‐determined sex are the result of a faulty PCR assay in these other populations. First, the ancestral *Amh08* locus amplified in 100% of our samples; thus, these primers are highly reliable for this locus. Although it is possible that the lack of amplification of *AmhY* in some males resulted from mutations in the primer binding sites, this is highly unlikely given that different primer sets were used to amplify the Alberta and Washington populations. Independent mutations in these different primer binding sites would have needed to occur, which is unlikely given the young age of the *AmhY* duplication. Finally, failure to amplify *AmhY* cannot explain why females with *AmhY* were identified. Thus, the imperfect association between genetic and phenotypic sex observed across all five populations is highly unlikely to result from a faulty genotyping assay.

## 4 Discussion

In brook stickleback, we observed an imperfect association between the genetic and phenotypic sex in several populations. The strength of this association varied between populations. Incomplete penetrance of genetic sex determination, sometimes referred to as “leaky” sex determination, is not a common finding, but has also been observed in common frogs ( *Rana temporaria* ) and tree frogs ( *Hyla arborea* ) in Europe (Dufresnes et al. 2014; Phillips et al. 2020). In these Ranid and Hylid frog examples, evidence for the leaky sex determination was first inferred from low sex chromosome differentiation, which likely results from X‐Y recombination in XY females (Perrin 2009; Rodrigues et al. 2018). Similar to our observations in brook stickleback, the extent of leakiness in sex determination varies among these Ranid and Hylid frog populations (Dufresnes et al. 2014, Phillips et al. 2020).

Our results, based only on observations of partial penetrance of the *AmhY* gene, do not allow us to test for the mechanism of the “leaky” sex determination that we observe here, but we discuss several possible causes below. The mismatches between field‐determined and assay‐determined sex observed in the present study may be due to feminizing or masculinizing environmental contaminants. In particular, endocrine‐disrupting compounds (EDCs) can act as agonists by mimicking hormones or as antagonists by blocking hormone receptors, interfering with hormone synthesis, or interfering with metabolism (Sumpter 2005; Evans et al. 2012). These contaminants can get into aquatic environments at low but biologically active concentrations from wastewater treatment plants (Jiang et al. 2005). Pharmaceuticals, such as oral contraceptives and therapy drugs, can also get into aquatic environments from wastewater treatment plants (Desbrow et al. 1998). For example, in the Oldman River in southern Alberta, Canada, synthetic estrogen has been found in the river water and has been causing an extreme level of feminization in longnose dace ( *Rhinichthys cataractae* ) to the point where, at some locations, 90% of the population has female reproductive tissue (Evans et al. 2012; Jeffries et al. 2010, 2008). Significant concentrations of hormones in freshwater systems have also been found to originate from the run‐off of agricultural lands, such as cattle farms (Orlando et al. 2004; Nemesházi et al. 2020) and fields that have been fertilized with chicken litter (Finlay‐Moore, Hartel, and Cabrera 2000). For example, in fathead minnows ( *Pimephales promelas* ), fish downstream of a cattle feedlot were masculinized by increased testosterone and androgen concentrations (Finlay‐Moore, Hartel, and Cabrera 2000). Interestingly, the medaka ricefish has been found to have a bidirectional response to a synthetic progestin called levonorgestrel (LNG), which is used in emergency contraceptives or birth control pills (Watanabe et al. 2023). LNG has also been found to have masculinizing effects in threespine stickleback (Svensson et al. 2013).

While environmental contaminants such as EDCs can clearly disrupt genetic sex determination in some cases, there are several reasons why we believe the mismatches between field‐determined sex and the presence‐absence of *AmhY* in brook stickleback observed in the present study are not driven by environmental contaminants. Firstly, Shunda Lake and Goldeye Lake are located near the headwaters in the Rocky Mountains and are upstream of any wastewater treatment plants or agricultural lands. Astotin Lake is located near the middle of Elk Island National Park (194 km^2^, established in 1913) far from urban or agricultural runoff, and the water quality in the lake is monitored monthly (Parks Canada 2022). Second, the presence of both male and female reproductive tissue in feminized longnose dace is a hallmark of EDC contamination, but we have never observed an individual with both male and female reproductive tissue in any brook stickleback population. Lastly, notwithstanding the bidirectional effects of LNG on medaka ricefish described above, the effects of environmental contaminants are typically unidirectional, causing either masculinization or feminization, whereas we have observed male brook stickleback without *AmhY* and female brook stickleback with *AmhY* in four of the five populations in our study.

Environmental stressors could have a role in the degree of “leakiness” in sex determination among the brook stickleback populations. Female‐to‐male sex reversal has been observed in Medaka fish *(Oryzias latipes)* in response to a number of environmental conditions, including temperature (Sato et al. 2005), hypoxia (Cheung, Chiu, and Wu 2014), starvation (Sakae et al. 2020), and the exposure to certain wavelengths of light (Hayasaka et al. 2019). If environmental stressors have a similar effect on brook stickleback in influencing sex determination, variation in local conditions among populations may explain the presence of phenotypic males without the *AmhY* duplication. Further work assessing levels of environmental contaminants and/or stressors in these populations are clearly needed to test these hypotheses.

Aside from the effects of environmental contaminants or stressors, we propose two additional hypotheses explaining the variation in sex determination in brook stickleback. Jeffries, Mee, and Peichel (2022) suggested that sex determination in brook stickleback might involve a dosage mechanism whereby increasing *Amh* expression via gene duplication such that when the amount of anti‐mullerian hormone (Amh) exceeds some threshold, male development is initiated. This dosage‐threshold sex determination system has been proposed for other species (Bachtrog et al. 2014). Our first hypothesis to explain our observation of variation in *AmhY* sex determination draws on the fact that gene expression is, inherently, a stochastic process (Raj and van Oudenaarden 2008; Beukeboom and Perrin 2014; Perrin 2016). For example, there might be variability in *Amh* expression, such that the threshold amount of Amh may be achieved in some individuals without *AmhY*, leading to males. Alternatively, the amount of Amh may not exceed the threshold in some individuals with *AmhY*, leading to females (Rodrigues et al. 2017). The differences between populations in the amount of “leakiness” in *AmhY* sex determination observed in our study (e.g., 44% mismatch in Astotin Lake versus 11% mismatch in Shunda Lake) would be explained, according to this threshold hypothesis, by differences in regulatory elements (i.e., enhancers and promoters) upstream of *AmhY*. There could also be allelic variants of the coding sequence of *AmhY* that render the protein non‐ or sub‐functional. A future direction we are exploring to address this hypothesis is, therefore, a comparison of the regulatory sequences upstream of *AmhY* as well as the coding region in all five populations to explore variability.

Our second hypothesis to explain our observation of inconsistent *AmhY* sex determination is that there is an additional locus somewhere in the brook stickleback genome that is also contributing to sex determination. Polygenic sex determination has been proposed in other species (Liew et al. 2012; Roberts et al. 2016; Ser, Roberts, and Kocher 2010; Vandeputte et al. 2007). Autosomal loci have also been documented in wild populations of Medaka that can induce the development of males despite having an XX genotype (Shinomiya et al. 2010). There may also be additional *Amh* duplications somewhere in the genome (i.e., in addition to the duplicated copy on chromosome 20), but preliminary evidence (i.e., unpublished data from early stages of a diploid brook stickleback genome assembly; M. A. White, personal communication) suggests that this is not the case. Furthermore, there is no evidence of additional copies of *Amh* among the three populations sequenced in Jeffries, Mee, and Peichel (2022).

The variation between brook stickleback populations in the extent of leakiness in the *AmhY* sex determination system is a particularly intriguing observation. The extent of leakiness in the Ranid and Hylid frog sex determination systems also varied among populations (Dufresnes et al. 2014; Phillips et al. 2020). In these frog systems, the extent of leakiness coincides with the phylogeography of the populations, such that populations located close to Pleistocene glacial refugia are more leaky (i.e., have less sex chromosome differentiation) than populations located further north, which would have been established more recently after post‐glacial range expansion (Dufresnes et al. 2014, Phillips et al. 2020). Similarly, in brook stickleback, the two Albertan populations at highest elevation and farthest from a putative Mississippian glacial refugium (Shunda Lake and Goldeye Lake), and likely colonized most recently, have the least leaky sex determination. A hypothesized mechanism underlying this phylogeographic correlate of leakiness is related to our first hypothesis proposed above. The populations close to the origin of post‐glacial range expansion (i.e., the glacial refugium) may have higher genetic diversity than populations in more recently colonized locations, which is a typical pattern in post‐glaciation landscapes such as North America and Europe (Hewitt 2001; Lessa, Cook, and Patton 2003). Higher genetic diversity may manifest in higher diversity of cis‐ and trans‐regulatory elements (e.g., enhancers and transcription factors), thereby adding to the variability among individuals in *Amh* expression and exacerbating the stochasticity in *Amh* expression that may be driving the leakiness in this system. Consistent with this hypothesis, Astotin Lake is the most genetically diverse and Shunda Lake is the least genetically diverse of the Albertan populations investigated in this study (Mee et al. 2024). We do not have a comparable estimate of genetic diversity for the Pine Lake population, but we note that this recently introduced invasive population likely has low genetic diversity due to founder effects, and it has one of the lowest rates of leakiness among the populations we studied—a pattern that is consistent with our hypothesis. Of note, a recent study of North American northern pike ( *Esox lucius* Linnaeus, 1758) found a similar pattern of less genetic sex determination (i.e., lack of a master sex‐determination gene) in less genetically diverse populations in more recently colonized areas following post‐glacial range expansion (Johnson et al. 2024).

The observed variability of sex determination within and between populations of brook stickleback suggests that sex determination in brook stickleback is more complex than initially thought (i.e., as per Jeffries, Mee, and Peichel 2022) and may still be evolving. Consequently, brook stickleback populations likely provide a rare opportunity to study the mechanisms that contribute to the diversity and evolution of genetic sex determination mechanisms. Future investigations of the factors driving the patterns reported in this study will, at the very least, inform our general understanding of the diversity of sex determination mechanisms.

## Author Contributions

**Grace C. Pigott:** conceptualization (supporting), data curation (lead), formal analysis (equal), investigation (lead), methodology (supporting), validation (lead), visualization (lead), writing – original draft (lead), writing – review and editing (supporting). **Marin E. Flanagan:** conceptualization (supporting), formal analysis (equal), investigation (equal), methodology (supporting), validation (equal), writing – review and editing (supporting). **Malcolm G. Q. Rogers:** data curation (supporting), formal analysis (equal), investigation (equal), writing – review and editing (supporting). **Massa Abo Akel:** formal analysis (equal), investigation (equal), writing – review and editing (supporting). **Erica G. Marlette:** formal analysis (equal), investigation (equal), writing – review and editing (supporting). **Matthew J. Treaster:** formal analysis (equal), investigation (equal), writing – review and editing (supporting). **Shannon K. Fox:** data curation (equal), formal analysis (equal), investigation (equal), methodology (equal), project administration (supporting), supervision (supporting), visualization (supporting), writing – review and editing (supporting). **Shaugnessy R. McCann:** formal analysis (equal), investigation (equal), writing – review and editing (supporting). **Catherine L. Peichel:** conceptualization (equal), funding acquisition (equal), methodology (lead), resources (equal), supervision (lead), writing – original draft (supporting), writing – review and editing (equal). **Michael A. White:** conceptualization (equal), funding acquisition (equal), methodology (equal), resources (equal), supervision (equal), writing – review and editing (equal). **Daniel L. Jeffries:** conceptualization (lead), formal analysis (equal), investigation (equal), methodology (equal), writing – original draft (supporting), writing – review and editing (equal). **Jonathan A. Mee:** conceptualization (supporting), data curation (supporting), formal analysis (supporting), funding acquisition (equal), investigation (supporting), methodology (equal), project administration (equal), resources (equal), supervision (lead), writing – original draft (supporting), writing – review and editing (lead).

## Conflicts of Interest

The authors declare no conflicts of interest.

## Data Availability

The entirety of the data for this study is presented in Tables 1 and 2 of this manuscript.
